# Isolation, Molecular Characterization and Antibiotic Susceptibility Pattern of *Vibrio parahaemolyticus* from Aquatic Products in the Southern Fujian Coast, China

**DOI:** 10.4014/jmb.2001.01005

**Published:** 2020-02-27

**Authors:** Yuanqing Hu, Fengxia Li, Yixian Zheng, Xinan Jiao, Liqing Guo

**Affiliations:** 1 School of Biological Science and Biotechnology, Minnan Normal University, Zhangzhou 363000, P.R. China; 2 Jiangsu Key Laboratory of Zoonosis, Yangzhou University, Yangzhou 5000, P.R. China; 3 Zhangzhou Center for Disease Control and Prevention, Zhangzhou 6000, P.R. China

**Keywords:** Multiple resistance, antimicrobial resistance genes, randomly amplified polymorphic DNA (RAPD), plasmid profiles

## Abstract

*Vibrio parahaemolyticus* is a major gastroenteritis-causing pathogen in many Asian countries. Antimicrobial resistance in *V. parahaemolyticus* has been recognized as a critical threat to food safety. In this study, we determined the prevalence and incidence of antimicrobial resistance in *V. parahaemolyticus* in the southern Fujian coast, China. A total of 62 isolates were confirmed in retail aquatic products from June to October of 2018. The serotype O3:K6 strains, the virulence genes *tdh* and *trh*, antibiotic susceptibility and molecular typing were investigated. Then plasmid profiling analysis and curing experiment were performed for multidrug-resistant strains. The results showed that the total occurrence of *V. parahaemolyticus* was 31% out of 200 samples. Five strains (8.1%) out of 62 isolates were identified as the *V. parahaemolyticus* O3:K6 pandemic clone. A large majority of isolates exhibited higher resistance to penicillin (77.4%), oxacillin (71%), ampicillin (66.1%) and vancomycin (59.7%). Seventy-one percent (44/62) of the isolates exhibited multiple antimicrobial resistance. All 62 isolates were grouped into 7 clusters by randomly amplified polymorphic DNA, and most of the isolates (80.6%) were distributed within cluster A. Plasmids were detected in approximately 75% of the isolates, and seven different profiles were observed. Seventy-six percent (25/33) of the isolates carrying the plasmids were eliminated by 0.006% SDS incubated at 42°C, a sublethal condition. The occurrence of multidrug-resistant strains could be an indication of the excessive use of antibiotics in aquaculture farming. The rational use of antimicrobial agents and the surveillance of antibiotic administration may reduce the acquisition of resistance by microorganisms in aquatic ecosystems.

## Introduction

*Vibrio parahaemolyticus* is a notorious marine organism that occurs naturally in estuaries and coastal regions throughout the world [1, 2]. Normally, *V. parahaemolyticus* bioaccumulates in the guts of filter-feeding molluscan shellfish, and it is a leading cause of seafood-associated gastroenteritis through the consumption of raw or undercooked aquatic foods [3, 4]. Antibiotics abuse has given rise to the global development and spread of drug resistance [1, 5, 6]. Clearly, *V. parahaemolyticus* can be a reservoir of resistance-associated genes and may play a role in the evolution and spread of antimicrobial resistance (AMR) in aquatic environments [1, 7].

AMR is a critical public health issue in coastal regions [1]. Antibiotics have been used in food animals for treatment, disease prevention, or growth promotion, and they have accelerated the spread of resistant bacteria and resistance genes from food animals to humans along the food chain [1, 5]. Furthermore, antimicrobial agents are released into marine aquaculture environments by agricultural runoff, providing opportunities for *V. parahaemolyticus* to acquire resistance mechanisms and subsequently concentrate in filter-feeding shellfish [6]. Unfortunately, the excessive and inappropriate use of antibiotics has resulted in the emergence of bacterial resistance to regular prescribed antibiotics, which can lead to failures in the available treatment options for common infections [8].

Molecular typing is important for assessing the transmission and diversity of strains and for differentiating between new infections and relapses [9]. All sorts of molecular typing tools have been used to study the prevalence of *V. parahaemolyticus*, and these methods include multi-locus sequence typing (MLST) [8, 10], pulsed-field gel electrophoresis (PFGE) [11], repetitive extragenic palindromic element-polymerase chain reaction (REP-PCR)[12], enterobacterial repetitive intergenic consensus (ERIC) [8, 13] and random amplified polymorphic DNA (RAPD) [14], etc. RAPD is useful for epidemiological studies because it provides adequate information on the source of infection, routes of transmission, suitable control measures, molecular markers of virulent and host-specific strains, and genetic relatedness of different bacterial isolates [1, 14, 15]. Moreover, RAPD is easier to perform, labor-saving, faster, highly reproducible and requires significantly less special equipment and reagents [16] compared with other methods, such as PFGE. RAPD can be used in molecular subtyping for *V. parahaemolyticus* independently or in combination with other typing methods [16, 17]. The RAPD profiles were determined for the 62 isolates of *V. parahaemolyticus* from the southern Fujian coast, and the relationship between drug resistance and genetic diversity was studied in this paper.

The coastal area of Fujian is important for the production and processing of aquatic products. In recent years, the *V. parahaemolyticus* epidemic has been more serious in this region, especially in summer and autumn. In aquaculture, the excessive use of antibiotic drugs is very common, and it causes the more serious problem of drug resistance in *V. parahaemolyticus*. To our knowledge, no similar studies have been reported along the southern Fujian coast, an important aquaculture area in China. In this paper, 62 strains of *V. parahaemolyticus* that were isolated from aquatic products were studied by molecular typing, drug resistance gene analysis, drug resistance observation and a clearance test for drug resistance plasmids to understand the resistance status of *V. parahaemolyticus*.

## Materials and Methods

### Bacterial Strains

From June to October of 2018, a total of 200 aquatic product samples were obtained, namely short-necked clam (*Paphia variegata*, *n* = 42), Asiatic hard clam (*Meretrix meretrix*, *n* = 36), razor clam (*Sinonovacula constricta*, *n* =34), oyster (*Crassostrea plicatula*, *n* = 45) and white shrimp (*Penaeus vannamei*, *n* = 43), which were collected from retail markets along the southern Fujian coast in China. The first three species are widely cultured in the southeastern coastal provinces of China, while the last two are grown along the southern Fujian coast. Suspected colonies were screened on thiosulfate-citrate-bile salts-sucrose (TCBS, Beijing Land Bridge Technology Co., Ltd., China) agar plates. Presumptive isolates of *V. parahaemolyticus* were confirmed by the presence of the species-specific gene *bla*CARB-17 [18] and *toxR* [19] genes using duplex PCR. The presumptive *V. parahaemolyticus* isolates were evaluated for the virulence genes *tdh* and *trh* using duplex PCR [20]. The reaction mixture for PCR assay was performed in 25 μl, which contained 12.5 μl of 2× Taq Master Mix (Shanghai Generay Biotech Co., Ltd., China), 1 μM of each primer, 3 μl of DNA template and the remaining volume of double-distilled water. The thermal-cycling program is as follows: initial denaturation at 95°C for 5 min; 30 cycles of 95°C for 30 sec, an annealing temperature of 58°C for 30 sec, and 72°C for 30 sec with a final extension of 72°C for 5 min. These positive strains were stored at -80°C for further analysis. The population numbers of *V. parahaemolyticus* in each aquatic sample were determined using the most probable number (MPN) method according to the Bacteriological Analytical Manual standard method and a previous study [4, 13, 21].

### Screening of Serovar O3:K6 by Triplex PCR

Triplex PCR was performed to detect the *toxR* gene specific to the *Vibrio* species, the serogroup O3-specific gene *vp0209* and the serovar K6-specific gene *vp0230* according to Zhang’s protocol [22-24]. The forward (F) and reverse (R) primers of the three detected genes are listed in Table 1. These oligonucleotide primers were synthesized by Sangon Biotech (China). The amplification conditions used for the triplex PCR were set up according to the protocol of Zhang *et al*. with slight modifications [24]. In brief, the 25 μl reaction mixture was composed of 12.5 μl of 2× Taq Master Mix (Shanghai Generay Biotech Co., Ltd.), each primer at 0.5 μM, 6.5 μL of ddH_2_O, and 2 μl of DNA template. The thermal cycling parameters for PCR were as follows: denaturation at 95°C for 2 min, 30 cycles of 95°C for 30 sec, an annealing temperature of 56°C for 30 sec, and 72°C for 1 min, with a final extension of 72°C for 5 min. PCR was performed on a Bio-Rad PTC-200 Thermal Cycler (Bio-Rad, USA). The amplified products were analyzed electrophoretically on a 1.2% agarose gel containing Gel Stain (Sangon Biotech, China) and photographed using an Amersham Imager 600 UV (GE Healthcare, USA).

### RAPD-PCR Genotyping

The randomly designed 9-mer oligonucleotide primer P1 (5’-AAGAGCCGT-3’) was used for RAPD typing as described previously by Hara-Kudo *et al*. [25]. The reactions were performed in 25 μl volumes consisting of 2.5 μl of 10× PCR buffer (Shanghai Generay Biotech Co., Ltd.), 1 μl of primer (50 pmol/μl), 2 μl of purified genomic DNA (50 ng/μl), 0.2 μl of the 10 mM dNTPs, 0.5 μl of Taq DNA polymerase (5 U/μl) and 18.8 μl of sterile distilled water. The following conditions were used for the amplification: initial denaturation at 94°C for 3 min, 40 cycles of denaturation at 94°C for 1 min, primer annealing at 36°C for 1 min and extension at 72°C for 2 min, with a final extension at 72°C for 7 min. The RAPD products were visualized using the same method as described above. A gel imaging map was analyzed using Gel Pro Analyzer 4.5 software, and the bands were marked as 0 (absence) or 1 (presence). Cluster analyses of RAPD data were performed using the unweighed pair group method of arithmetic averages (UPGMA) and NTSYS-pc (Version 2.10, http://www.exetersoftware.com/cat/ntsyspc.html).

### Antimicrobial Susceptibility Testing

The antibiotic susceptibility of the 62 isolates was tested using the disk diffusion method on Mueller-Hinton agar (MHA) (OXOID Limited, China) in accordance with the guidelines by the Clinical and Laboratory Standards Institute (CLSI, 2015). Thirty selected antimicrobials belonging to 11 classes used in this study were as follows: β-lactam (piperacillin: TZP, carbenicillin: CAR, oxacillin: OX, ampicillin: AMP, piperacillin: PRL, ceftazidime: CAZ, cefradine: CE, cephalexin: CL, cefazolin: KZ, cefuroxime: CXM, cefoperazone: SCF, cefatriaxone: CRO), aminoglycoside (gentamicin: CN, kanamycin: K, amikacin: AM, neomycin: N), macrolide (erythromycin: E, maddie mycin: MAG), tetracycline (tetracycline: TET, minocycline: MH, doxycycline: DO), quinolone (ciprofloxacin: CIP, norfloxacin: NOR, ofloxacin: OFLX), glycopeptide (vancomycin: VA), chloramphenicol (chloramphenicol: C), nitrofurans (furazolidone: FT), sulfonamides (trimethoprim-sulfamethoxazole: SXT), polypeptide (polymyxin B: PB), and lincosamides (clindamycin: DA). The results were expressed as sensitive (S), intermediate (I), or resistant (R) according to the method by the Clinical and Laboratory Standards Institute (CLSI, 2015). *Escherichia coli* ATCC 25922 was used as the reference strain for the antimicrobial susceptibility detection. The antimicrobial resistance of all the isolates was performed with three repetitions.

### MAR Index and Multidrug-Resistant (MDR) Definition

 The MAR index was created by Krumperman (1983), and it was calculated using the formula a/b where ‘a’ represents the number of antibiotics to which the specific isolate is drug-resistant, and ‘b’ represents the total number of multiple antibiotics to which the specific isolate has been exposed [26]. MDR was defined as the nonsusceptibility of the organism to at least one agent in three or more categories of antimicrobials. In our study, the MAR and MDR were calculated according to the above definition [26].

### Evaluation of Antibiotic Resistance-Encoding Genes

The 19 genes with antibiotic resistance to 7 classes of antibiotics were analyzed by PCR method. The 19 antibiotic resistance genes were associated with β-lactam (*bla*TEM, *bla*OXA, *bla*SHV, and *bla*CTX-M), Aminoglycoside (*strA* and *strB*), Tetracycline (*tetA*, *tetB*, and *tetM*), Chloramphenicol (*cat*I, *cat*II, *cat*III, and *cat*IV), Quinolone resistant determinant region (*gyrA* and *parC*), integron (*intI1*, *intI2*, and *intI3*) and the class I integron variable region gene box. The primers for the antibiotic resistance genes and the PCR conditions are shown in Table 1. The amplified products were analyzed using the same method as described above.

### Plasmid Profiling Analysis

The multiple drug resistant strains were evaluated for the presence of plasmids. Plasmid extraction was performed using the centrifugal column type of plasmid preparation kit method (Generay Biotech.). Electrophoretic separation was performed at 80 V for 50 min and a molecular weight marker DL2000 (Bioteke, China) was included. The gels were visualized under a UV transilluminator and recorded as TIFF files using a Gel Documentation System (GelDoc EZ imager, Bio-Rad).

### Plasmid Curing Experiments

The plasmids were cured for the isolates that were resistant to more than one antibiotic. The protocol was performed using Luria-Bertani broth supplemented with 3% NaCl and 0.006% sodium dodecyl sulfate (SDS) at a 42°C sublethal condition [27]. The plasmid DNA were extracted using a TIANprep Mini Plasmid Kit II (TIANGEN BIOTECH CO., LTD China). The plasmid pattern analysis was visualized using the same method as described above. After the plasmid was successfully cleared, the resistance of these strains was tested again by disk diffusion method.

## Results

### Prevalence and Density of *V. parahaemolyticus* in Aquatic Products

**Fig. 1:**
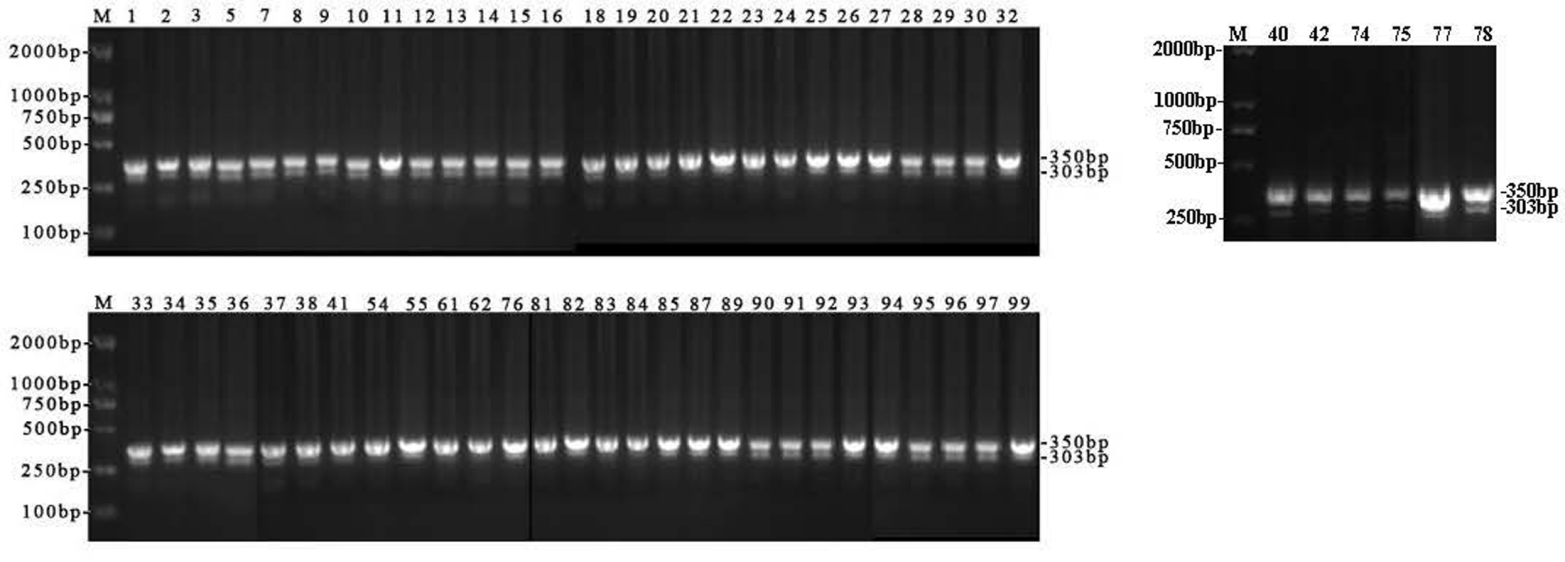
Agarose gel (1.2%) electrophoresis showing the results of duplex PCR amplification for the *bla*CARB- 17 and *toxR* genes of 62 *V. parahaemolyticus* isolates. The amplified bands show the expected molecular size of 303 bp for the *bla*CARB-17 gene and 368 bp for the *toxR* gene. M: DNA size markers; lanes with arabic numbers are the isolates of *V. parahaemolyticus*.

**Fig. 2:**
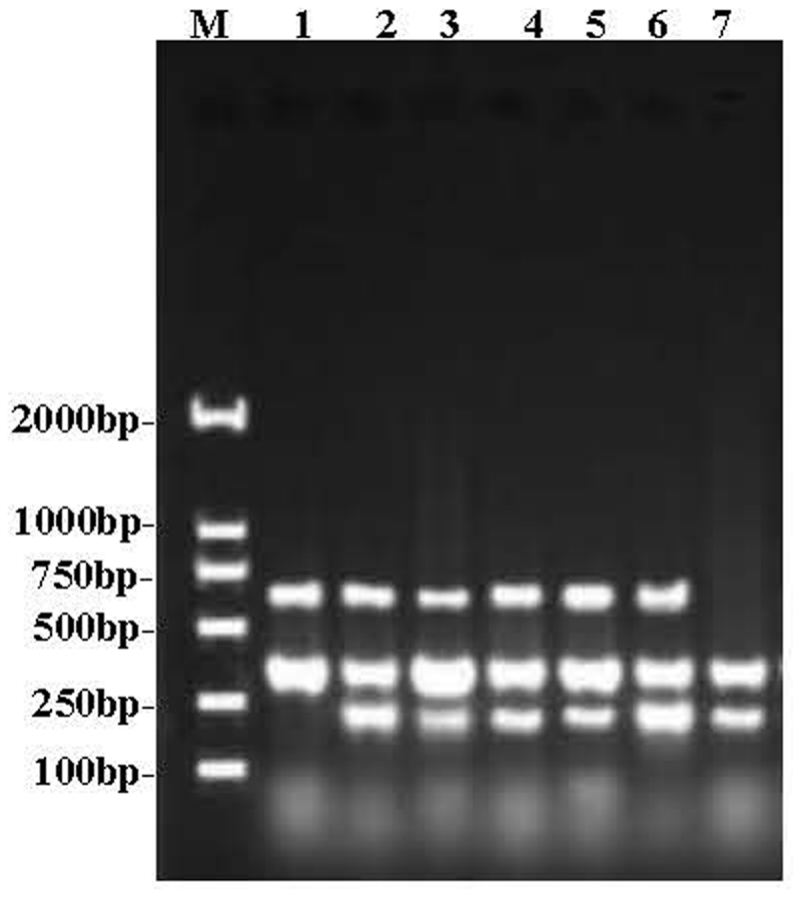
Agarose gel (1.2%) electrophoresis showing the results of triplex PCR amplification for screening of the O3:K6 serovar in 62 *V. parahaemolyticus* isolates. Amplified bands with the expected molecular size of 240 bp for the *vp0209* gene, 368 bp for the *toxR* gene and 623 bp for the *vp0230* gene. The O3 serovar was identified during the detection of the *vp0209* and *toxR* genes. The K6 serovar was identified when detecting the *vp0230* and *toxR* genes. Strains of serotype O3:K6 were confirmed when the 3 genes were detected simultaneously. Lane M, DL 2000 DNA marker; Lane 1, K6 serotype strain; Lane 7, O3 serotype strain; Lane 2-6, O3:K6 serotype isolates.

*V. parahaemolyticus* is one of the major food-borne bacteria and is frequently isolated from various aquatic products. From June to October 2018, we tested 200 aquatic food samples from 10 retail markets along the southern Fujian coast. The prevalence of *V. parahaemolyticus* in different samples is shown in Table 2. Overall, *V. parahaemolyticus* was detected in 76 (38%) of the 200 samples identified by traditional microbiological method, including the appearance of green colonies on TCBS agar and microscopic examination (gram-negative, curved rod-shaped, nonspore-forming bacilli), which are consistent with the national food safety standard of microbiological examination of food *V. parahaemolyticus* (GB4789.7-2013). Using duplex PCR for the species-specific *toxR* and *bla*CARB-17 genes, 62 of the 200 samples (31%) were determined to be positive for the presence of *V. parahaemolyticus*. The target bands of 303 bp (*bla*CARB-17) and 368 bp (*toxR*) were obtained by PCR amplification and gel electrophoresis (Fig. 1). All 62 isolates showed the normal biochemical characteristics of *V. parahaemolyticus*. These 62 isolates were the sum of 9 (21%), 7 (19%), 12 (35%), 29 (64%), and 5 (12%) isolates as collected from the short-necked clam, Asiatic hard clam, razor clam, oyster and white shrimp, respectively (Table 2). Three isolates containing the *tdh* gene were obtained from oyster samples, and no isolates with the *trh* gene were found. In our study, the pandemic clone O3:K6 serotype was determined by triplex PCR method. Five isolates were identified as strains of serovar O3:K6, which carry the specific genes *vp0209*, *vp0230*, and *toxR* (Fig. 2). The densities were <10 MPN/g in 61.3% (38/62) of the *V. parahaemolyticus* isolates. However, the densities of the remaining positive samples (24/62) ranged between 10 and 100 MPN/g (Table 2). According to the national food safety standard limit of pathogenic bacteria in food (GB29921-2013), these results of MPN are the acceptable level of pathogenic bacteria in China.

### RAPD Typing

**Fig. 3:**
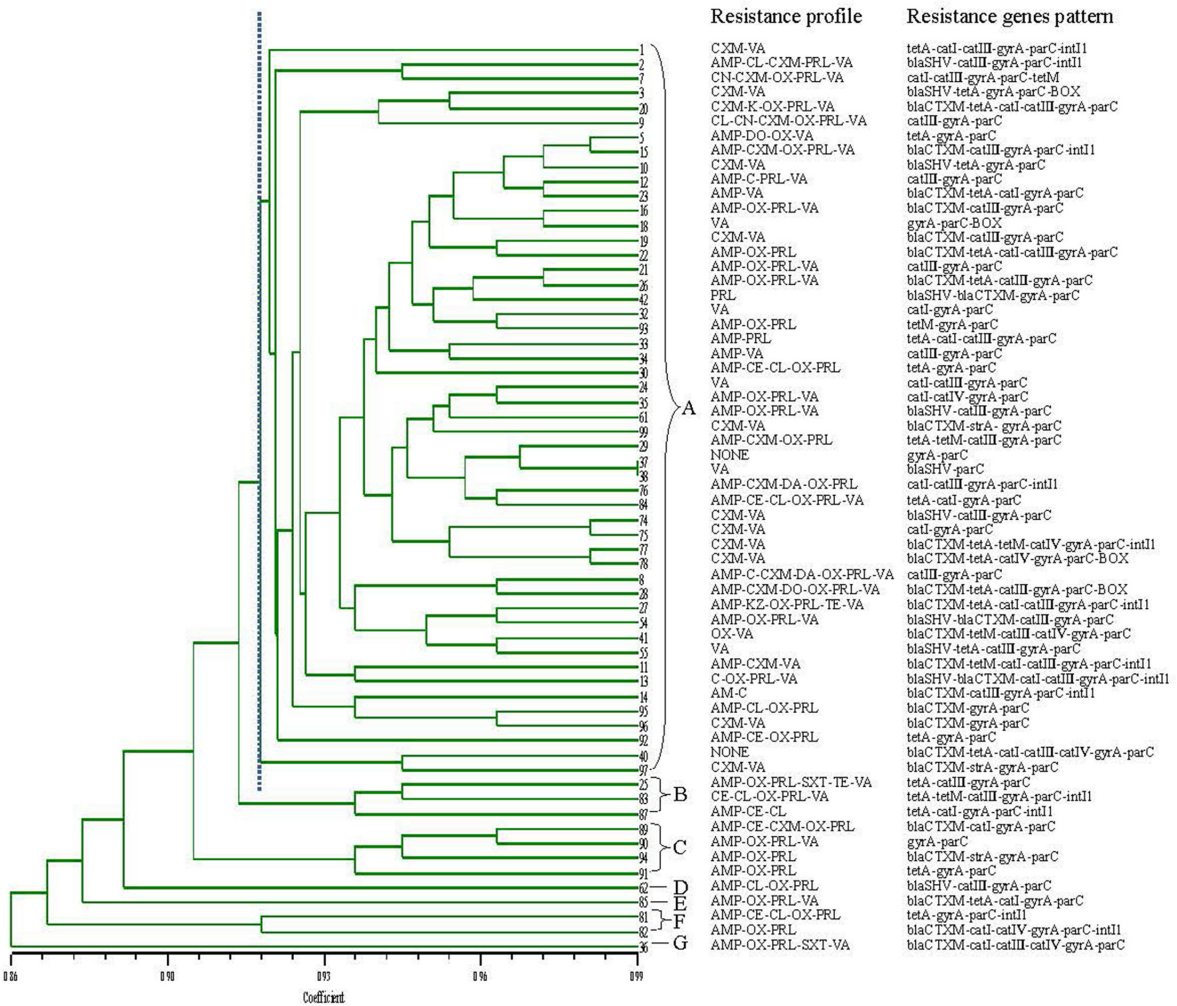
The relationships the genetic diversity, resistance phenotype and genotype characterization of 62 *V. parahaemolyticus* isolates. A phylogenetic tree of different *V. parahaemolyticus* isolates was drawn based on the RAPD results. A cluster analysis was performed using NTSYS-pc software based on the Dices similarity coefficient (SD) using a 1% position tolerance and the UPGMA.

In this study, a total of 62 isolates of *V. parahaemolyticus* were analyzed by RAPD to estimate their genetic relationship. The results of RAPD fingerprinting showed strong genetic diversity (Fig. 3). The DNA profiles from the RAPD exhibited 3-10 amplified bands ranging from 180 bp to approximately 4,000 bp. After hierarchical clustering analysis with the UPGMA, these polymorphic patterns were divided into 7 clusters (designated as A-G). Most isolates (80.6%, 50/62) were distributed in cluster A. There is only one strain in three clusters known as D (62#), E (85#) and G (36#). The three isolates (25#, 83#, and 87#) were distributed within the B cluster. The four isolates (89#, 90#, 91#, and 94#) were distributed within the C cluster. Two isolates (81# and 82#) belonged in cluster F. The results show that the typing is correlated with the geographical distribution of the samples. The isolates from the white shrimp samples collected from Yunxiao were primarily grouped into B, E, F, and G. Most of the *V. parahaemolyticus* isolated from Zhangzhou city were grouped into cluster A, but the other 4 strains were grouped into clusters B and C. One strain of white shrimp from Longhai was grouped into cluster D only. The strains isolated from Dongshan were not grouped into the same cluster as those from the closer location of Yunxiao. Instead, these strains were grouped into cluster A with those isolated from Zhangzhou City.

### Antimicrobial Susceptibility

Thirty antimicrobials, including β-lactams, quinolones, aminoglycosides, tetracyclines, macrolides, lincosamides, chloramphenicols, sulfonamides, glycopeptides and polymyxins, were chosen for antimicrobial susceptibility testing, and the susceptible, intermediate and resistance rates are shown in Table 3. The antimicrobial susceptibility testing ranges are also presented. None of the 62 isolates were resistant to CAZ, SCF, CRO, TZP, CIP, NOR, OFLX, and E, and all the strains were sensitive to these drugs. The rates of resistance to AMP, OX, PRL, and VA reached 66.1%, 71%, 77.4%, and 59.7%, respectively. Smaller percentages of the isolates were resistant to CXM, CE, CL, CN, DO, TE, DA, C, and SXT, and the rates of resistance were 19.4%, 17.7%, 17.8%, 4.8%, 8.1%, 6.5%, 8.1%, 11.3%, and 6.5%, respectively. By contrast, a large number of isolates had intermediate resistance to AM (67.7%), CXM (80.6%), CE (79%), KZ (79%), CL (82.3%), MH (93.5%), N (80.6%), DO (91.9%), and TE (72.6%). All the isolates showed intermediate resistance to K.

The MAR index was evaluated by finding the ratio of the number of antibiotics to which the organism was resistant to the total number of antibiotics used [8, 28]. The MAR index, which was first proposed by Krumperman (1983), represents the extent of environmental contamination by antimicrobials, and it is used to assess the potential risk to human health. When an MAR index exceeds 0.2, aquatic foods are considered as high-risk sources of antimicrobial contamination. In the present study, 71% (44/62) of the *V. parahaemolyticus* isolates exhibited multiple antimicrobial resistance to at least 2 antimicrobials. The MAR index of most isolates was between 0.07 and 0.23 (Table 4). The maximum MAR index was attributed to one isolate that was resistant to 7 antibiotics. The present study found 11 out of 44 (25%) multidrug-resistant strains of *V. parahaemolyticus* to be MDR. One of the serotype O3:K6 isolates was MDR. No correlation was found between the molecular typing and drug resistance spectrum of these strains.

### Antimicrobial Resistance Genotypes of *V. parahaemolyticus*

Nineteen antimicrobial resistance genes belonging to 7 classes detected in 62 *V. parahaemolyticus* isolates are shown in Table 5 and Fig. 3. The PCR method used to detect the occurrence of antimicrobial resistance genes (ARGs) was based on the genomic DNA of 62 isolates. Seven out of 19 resistance genes (*bla*TEM, *bla*OXA, *strB*, *tetB*, *cat*II, *intI2*, and *intI3*) were not detected in all the isolates. Other 12 genes were detected in one or more isolates. Clearly, all of the isolates carried two or more detected ARGs. Among these evaluated genes, *parC* was the most prevalent gene, with detection rate of 100%, followed by *gyrA*, *cat*III, *bla*CTX-M, *tetA*, *cat*I, *intI1*, *bla*SHV, *tetM*, *cat*IV, BOX and *strA*, and the detected percentages of them were 98.4%, 54%, 42.9%, 41.3%, 33.3%, 20.6%, 17.5%, 11.1%, 11.1%, 9.5%, and 5.8%, respectively.

### Plasmid Profiling and the Result of Plasmid Curing in the Isolates

Plasmid profiles of the 44 multiple drug resistant isolates were performed. Plasmids were found in 75% (33/44) of the isolates and 7 different plasmid profiles (P1-P7) were observed (Table 6). No plasmid was detected in 11 isolates. Plasmid profiles 1 (P1: 33 kb) and 2 (P2: 33 kb, 15 kb) were the most common profiles among the isolates from aquatic products. The plasmids were eliminated by 0.006% SDS combined with a 42°C sublethal condition. Twenty-five of the 33 strains carrying the plasmid were not detected after the plasmid curing, and the elimination rate reached 76%. The drug resistance of the strains from which the plasmids were cleared also changed. Isolates 11# and 82# became moderately resistant to ampicillin, 20# and 76# became moderately resistant to cefuroxime, 14# became susceptible to amikacin, 20# became susceptible to kanamycin, 13# became susceptible to oxacillin, and 82# became susceptible to penicillin from drug resistance.

## Discussion

*V. parahaemolyticus* is considered as an important seafood-borne pathogen because of its involvement in outbreaks after the consumption of contaminated foods [29]. In recent decades, the antibiotics used in aquaculture have been largely used for therapeutic and prophylactic purposes, and antibiotic resistance is a global public health concern and a food safety problem [30]. In the present study, we analyzed 200 aquatic product specimens that were collected from the retail markets along the southern Fujian coast from June to October of 2018 and isolated 62 *V. parahaemolyticus* strains, which had a positive rate of 31%, suggesting the possibility that there were gastroenteritis cases associated with the consumption of uncooked seafood or cross-contaminated food in coastal areas during warmer months. Compared to other studies in China in recent years, this positive rate is slightly lower than the 47.2% reported for Jiangsu Province [14], the 38.0% reported in Zhejiang Province from 2010 to 2014 [31], the 43.75% observed from the markets of 11 cities in four provinces of South China [13] and the 34.3% reported in Shanghai from February 2014 to February 2015 [8]. Compared to other countries, our result is also significantly less than the 83% rate in raw oysters in Brazil [20], 67% in fish in Jordan [19] and 80.8% in shrimp in Ecuador [32]. This finding indicates that the current status of *V. parahaemolyticus* prevalence in the southern Fujian coast is better than that of other regions. In our study, the positive rate of *V. parahaemolyticus* in oysters was 64%, which was significantly higher than that of other aquatic samples. Because oysters need to filter more seawater to provide essential nutrition by eating organic matter from the surrounding water, oysters simultaneously bioaccumulate more pathogenic organisms from water than other shellfish [8]. The positive isolates were confirmed by duplex PCR based on the detection of the *bla*CARB-17 and *toxR* genes, and ultimately, 62 out of 76 *V. parahaemolyticus* strains were obtained. The above results are also consistent with the biochemical experiments and Gram staining test. Although many of the targeting genes (*tlh* and *atpA*) were used as species-specific markers, some of them may share similar sequences within the *Vibrio* species, reducing the accuracy and specificity of these detection methods [18, 33]. A novel *bla*CARB-17 gene was identified and found to be responsible for the intrinsic penicillin resistance in *V. parahaemolyticus* [18, 34]. Li *et al* analyzed the homologies of the *tlh*, *atpA* and *bla*CARB-17 genes, and the last is more highly conserved than the other two genes [18]. The specificity of the PCR based on the *bla*CARB-17 gene is 100%, whereas the PCR targeting the *tlh* and *atpA* genes occasionally produced false positive results [18]. To improve the detection accuracy, a duplex PCR targeting the *toxR* and *bla*CARB-17 genes was developed and used to identify the *V. parahaemolyticus* in our study. Here, 3 (4.8%) isolates were *tdh*-positive *V. parahaemolyticus*, whereas no *trh*-positive strains were found. The finding of fewer *tdh*- and/or *trh*-positive strains of *V. parahaemolyticus* indicates lower aquatic food risk. The pandemic O3:K6 serovars are considered possibly responsible for recent *V. parahaemolyticus* outbreaks in many countries, and the majority of infections are acquired by the means of polluted seafood [35, 36]. In the present study, 5 isolates were identified as the serovar O3:K6 clone by triplex PCR method. These findings contrast with the results of a previous study [37, 38].

The RAPD assay has been proved to be a powerful tool for discriminating among different species or subspecies and for the genetic analysis of phylogenetic relationships among strains or populations for a variety of microorganisms [14]. Yang *et al* assessed the genetic diversity among 251 isolates, and 33 different genetic patterns were grouped into 9 clusters at an 82% similarity level by RAPD method [39]. Chao *et al* identified 341 strains of *V. parahaemolyticus*, which were clustered into six molecular types and 23 subtypes using the RAPD analysis [14]. Zhao *et al* analyzed the genetic diversity of 157 isolates, and 73 different patterns were identified, which were grouped into 18 clusters [16]. Compared with pulsed-field gel electrophoresis (PFGE), enterobacterial repetitive intergenic consensus sequence PCR (ERIC-PCR) and repetitive extragenic palindromic sequence PCR (REP-PCR), RAPD-PCR is superior in that it is completed more quickly, is cost-effective, less labor-intensive and is based on differences in nucleotide sequences in the whole genome [39]. The RAPD method has been used successfully for subdividing *V. parahaemolyticus* from different seafood in India and for other organisms [40-43]. Selecting effective primers is key to the successful implementation of RAPD. In reference to Chao *et al* 2009, we chose P1 as the amplification primer and obtained better RAPD patterns [14]. In this study, all 62 isolates were identified with individual patterns. These patterns were grouped into 7 RAPD clusters with a coefficient of more than 0.918, and cluster A is predominantly present in the southern Fujian coast, China. These isolates (80.6%) were grouped into the same RAPD cluster A but did not show identical RAPD patterns, suggesting that these isolates were genetically very closely related.

*Vibrio* is the most important pathogenic bacteria in aquaculture, and it often causes serious diseases and results in serious economic losses [44, 45]. The global increase in the prevalence of antibiotic-resistant *Vibrio* is a major concern for food safety and fish farming [46]. Most of the tested antibiotics, such as ciprofloxacin, chloramphenicol, gentamicin and ceftazidime, are recommended as treatments for *Vibrio* infection [2, 3]. In the current study, 77.4% and 71% of the isolates showed resistance to penicillin and oxacillin, respectively, and these results were comparable to data obtained in other countries. Ceftazidime, cefoperazone, cefatriaxone, piperacillin, ciprofloxacin, norfloxacin, ofloxacin and erythromycin were found to be the most efficient at eliminating *V. parahaemolyticus* strains isolated from aquatic products along the southern Fujian coast of China (Table 3). The isolates in our collected samples were most sensitive to ceftazidime, cefoperazone, cefatriaxone, piperacillin, ciprofloxacin, norfloxacin, ofloxacin and erythromycin. This finding was satisfactory because these antibiotics are the preferred drugs for treating infections caused by *Vibrio* spp. [47]. The efficiency of ceftazidime, cefoperazone, cefatriaxone and piperacillin is related to their ability to inhibit the mucopeptide synthetase of the cell wall [48]. Thus, the synthesis of cell wall mucopeptides is blocked, the bacterial cell wall is defective, and the bacterial body expands and splits. To our knowledge, quinolones, tetracyclines and sulfonamides are widely used in coastal aquaculture in China. In our study, all 62 isolates were sensitive to ciprofloxacin, norfloxacin and ofloxacin. However, high incidences of intermediate resistance to minocycline (93.5%) and furazolidone (45.2%) were found. Similarly, most strains showed moderate tolerance to tetracyclines, including doxycycline and tetracycline, and the incidences of intermediate resistance are 91.9% and 72.6%, respectively. Only a small number of strains showed resistance and moderate tolerance to sulfonamides, at 8.1% and 16.1%, respectively. Notably, the results showed that 77.4% of the isolates were resistant to PRL. A higher percentage of antibiotic resistance was also found in AMP (66.1%), OX (71%) and VA (59.7%). These results were partially consistent with other studies performed in China [6,8,49-51]. Surprisingly, isolates of *V. parahaemolyticus* were resistant to the first-generation cephalosporins (CE 17.7% and CL 17.8%), but no resistance profile was observed within the isolates towards the third-generation cephalosporins (CAZ, SCF, and CRO). This finding is primarily due to the abuse of the first-generation cephalosporins over the past few decades. In summary, the strains that are resistant to quinolones, tetracyclines and sulfonamides are not particularly serious in this region. There is also a relatively high proportion of medium-resistant strains, and they may be transformed into drug-resistant strains under environmental stress. The high prevalence of antimicrobial resistance among seafood-derived *V. parahaemolyticus* is a risk to human health, and the resistant isolates might be a potential source of risk for the storage and transmission of resistance genes. This problem must be monitored by the relevant departments.

More than half (71%) of the *V. parahaemolyticus* isolates demonstrated MAR to at least two antimicrobials (Table 4), with a MAR range of 0.07 to 0.23 and an average of 0.14. The MAR index was as high as 0.23 in one isolate (8#) that was resistant to seven antibiotics. The variation in the MAR index could be attributed to differences in the sources of samples, geographic distribution and test methodologies [9, 52]. Among these factors, diverse geographical environments had differential selective pressures for the antibiotic resistance levels [9]. In Korea, high MAR indexes of 0.38 and 0.75 were found in *V. parahaemolyticus* strains from oyster samples [3]. In Malaysia, an MAR index of 0.93 was found in *V. parahaemolyticus* isolates from fish samples [44]. A high MAR index (0.63) was reported in *V. parahaemolyticus* isolates from environmental samples in England [53]. In this study, the MAR index of almost half of the isolates was less than 0.2, suggesting that most of the seafood presented lower risks of antimicrobial contamination in this region. *V. parahaemolyticus* lives in coastal and estuarine waters, which are particularly subject to environmental contamination by agricultural runoff or effluent from wastewater treatment plants. Aquatic bacteria suffer from selective pressure for antimicrobial resistance from the various levels of antimicrobials and heavy metals in the estuarine or offshore environments [54].

Among the 3 tetracycline resistance genes evaluated in our study, the detection frequencies of *tetA* and *tetM* were approximately 41.3% and 11.1%, respectively. The *tetA* gene was found in *Vibrio* cholera isolates collected from shrimp ponds in Thailand [55]. Another tetracycline resistance gene, *tetB*, was not detected in this study. The aminoglycoside resistance gene *strA* was detected in 5.8% of the isolates and *strB* was not found. these results are not consistent with the findings by Lou *et al*. 2016 [2]. Two quinolone resistance genes evaluated here, *gyrA* and *parC*, were detected in 98.4% and 100% of the isolates, respectively, and they are normally detected in bacteria. Our findings demonstrated that isolates possessing resistance genes might not be resistant to the relevant antimicrobial agents. This study is the first large-scale survey on the antimicrobial resistance genes of seafood-derived *V. parahaemolyticus* isolates from the southern Fujian coast, China.

Plasmids are considered one of the most significant mediators of the speedy spread of antibiotic resistance within bacteria [56], so it is essential to screen genetic elements such as the plasmids associated with antibiotic resistance in microorganisms. In our study, we were able to detect the presence of plasmids within 75% of the isolates. Seven different plasmid patterns were shown. A 33 kb plasmid was found in all 44 multiple drug resistance isolates, indicating the presence of 30 kb plasmids in environmental *Vibrio* isolates, which is consistent with the findings of Zhang *et al*. [57]. As previously shown, bacterial antibiotic resistance profiles are associated with large plasmids and their conjugation [56]. In our study, no correlation was found between the antibiotic resistance patterns and the plasmid profiles.

In conclusion, we present a comprehensive study involving molecular typing, antimicrobial susceptibility, resistance-associated genotypes and plasmid profiles of *V. parahaemolyticus* isolated from aquatic products collected along the southern Fujian coast of China from June to October of 2018. The occurrence of *V. parahaemolyticus* was 31%, which represents a decrease compared to other previous reports. The virulence gene *tdh* was detected in three isolates (4.8%), and the O3:K6 pandemic clone was determined in five strains (8.1%). In addition, the results showed that 77.4% of the *V. parahaemolyticus* was resistant to PRL, and resistance was also shown to OX, AMP and VA. Notably, a large majority of isolates (71%) exhibited multiple antimicrobial resistance. However, there is no apparent correlation among the resistance phenotype, genotype and plasmid patterns in our study. Given the complex situation of antimicrobial resistance in aquatic food, broader monitoring and investigation are needed in China.

## Figures and Tables

**Table 1:** Oligonucleotide primers used in this study.

Target genes	Primer	Sequence (5' to 3')	Amplicon size (bp)	Annealing temperature (℃)
*bla*CARB-17	CARB-F	ACC(T)TTGATGGAAGATA	303	58
	CARB-R	T(C)TAACTTTCTTTGTAGTGC(A)		
*toxR*	toxR-F	GTCTTCTGACGCAATCGTTG	368	58
	toxR-R	ATACGAGTGGTTGCTGTCATG		
*tdh*	tdh-F	GTAAAGGTCTCTGACTTTTGGAC	269	58
	tdh-R	TGGAATAGAACCTTCATCTTCACC		
*trh*	trh-F	TTGGCTTCGATATTTTCAGTATCT	500	58
	trh-R	CATAACAAACATATGCCCATTTCCG		
RAPD	random site	AAGAGCCGT	random size	36
*bla*TEM	blaTEM-F	ATAAAATTCTTGAAGACGAAA	1086	58
	blaTEM-R	GACAGTTACCAATGCTTAATC		
*bla*OXA	blaOXA-F	ACACAATACATATCAACTTCGC	885	60
	blaOXA-R	AGTGTGTTTAGAATGGTGATC		
*bla*SHV	blaSHV-F	GCGAAAGCCAGCTGTCGGGC	304	58
	blaSHV-R	GATTGGCGGCGCTGTTATCGC		
*bla*CTX-M	blaCTX-M-F	GTGCAGTACCAGTAAAGTTATGG	538	58
	blaCTX-M-R	CGCAATATCATTGGTGGTGCC		
*strA*	strA-F	CCTGGTGATAACGGCAATTC	546	58
	strA-R	CCAATCGCAGATAGAAGGC		
*strB*	strB-F	ATCGTCAAGGGATTGAAACC	509	54
	strB-R	GGATCGTAGAACATATTGGC		
*tetA*	tetA-F	GCTACATCCTGCTTGCCTTC	210	46
	tetA-R	CATAGATCGCCGTGAAGAGG		
*tetB*	tet B-F	TTGGTTAGGGGCAAGTTTTG	659	55
	tetB-R	GTAATGGGCCAATAACACCG		
*tetM*	tetM-F	GTRAYGAACTTTACCGAATC	633	52
	tetM-R	ATCGYAGAAGCGGRTCAC		
*catⅠ*	C-1	GGTGATATGGGATAGTGTT	349	57.5
*catⅡ*	C-2	GATTGACCTGAATACCTGGAA	567	54
*catⅢ*	C-3	CCATACTCATCCGATATTGA	275	54
*catⅣ*	C-4	CCGGTAAAGCGAAATTGTAT	451	60.5
Cat reverse universal primer	C-R	CCATCACATACTGCATGATG	-	-
*gyrA*	gyrA-F	CGATTGGAACAAACCATATAAA	200	56
	gyrA-R	CGGTGTAACGCATTGCCGCA		
*parC*	parC-F	CTTGGTCTTTCGGCATCAGC	214	56
	parC-R	CTTCGGTATAACGCATTGCC		
*intI1*	IntI1-F	GGGTCAAGGATCTGGATTTCG	483	58
	IntI1-R	ACATGCGTGTAAATCATCGTCG		
*intI2*	IntI2-F	CACGGATATGCGACAAAAAGGT	788	60.8
	IntI2-R	GTAGCAAACGAGTGACGAAATG		
*intI3*	IntI3-F	GCCTCCGGCAGCGACTTTCAG	979	60.8
	IntI3-R	ACGGATCTGCCAAACCTGACT		
Gene cassette	Hep58	TCATGGCTTGTTATGACTGT	random size	45
	Hep59	GTAGGGCTTATTATGCACGC		
*vp0230*	VP-0230F	TCCTGTTGTGATAAAGTTGGCATT	623	56
	VP-0230R	CCGAATCAAGAACTAACCCACA		
*vp0209*	VP-0209F	GCATCAACCCATTTCAACTT	240	56
	VP-0209R	TTCCATACTTGGGTTGAGTTTTC		

**Table 2:** Prevalence of *V. parahaemolyticus* in different samples collected in southern Fujian coast, China.

Sample category	Number of samples collected	Positive rate	Positive number	Number of samples containing *V. parahaemolyticus* (MPN/g)	

				<10	10-10^2^
Short-necked clam	42	21%	9	5	4
Asiatic hard clam	36	19%	7	4	3
Razor clam	34	35%	12	7	5
Oyster	45	64%	29	19	10
White shrimp	43	12%	5	3	2
Yotal	200	31%	62	38	24

**Table 3:** Results of antibiotic susceptibility tests on 62 *V. parahaemolyticus* isolates from aquatic products.

Antimicrobial class	Antimicrobial agents	*V. parahaemolyticus* (*n* = 62)			Zone diameters (mm)		
	
		No. (%) of R	No. (%) of I	No. (%) of S	R	I	S
β-lactams	Amikaein (AM)	0 (0.0)	42 (67.7)	20 (32.3)	≤13	14-17	≥18
	Ampicillin (AMP)	41 (66.1)	21 (33.9)	0 (0.0)	≤13	14-16	≥17
	Carboxybenzicillin (CAR)	0 (0.0)	24 (38.7)	38 ( 61.3)	≤14	14-16	≥17
	Ceftazidime (CAZ)	0 (0.0)	0 (0.0)	62 (100.0)	≤14	15-17	≥18
	Cefoperazone (SCF)	0 (0.0)	0 (0.0)	62 (100.0)	≤15	16-20	≥21
	Cefatriaxone (CRO)	0 (0.0)	0 (0.0)	62 (100.0)	≤15	16-20	≥21
	Cefuroxime (CXM)	12 (19.4)	50 (80.6)	0 (0.0)	≤14	15-17	≥18
	Cefradine (CE)	11 (17.7)	49 (79.0)	2 (3.2)	≤14	15-17	≥18
	Cefazolin (KZ)	0 (0.0)	49 (79.0)	13 (21.0)	≤14	15-17	≥18
	Cephalexin (CL)	11 (17.8)	51 (82.3)	0 (0.0)	≤14	15-17	≥18
	Oxacillin (OX)	44 (71.0)	18 (29.0)	0 (0.0)	≤10	11-12	≥13
	Piperacillin (TZP)	0 (0.0)	0 (0.0)	62 (100.0)	≤17	18-20	≥21
	Penicillin (PRL)	48 (77.4)	14 (22.6)	0 (0.0)	≤19	-	≥20
Quinolones	Ciprofloxacin (CIP)	0 (0.0)	0 (0.0)	62 (100.0)	≤15	16-20	≥21
	Norfloxacin (NOR)	0 (0.0)	0 (0.0)	62 (100.0)	≤12	13-16	≥17
	Ofloxacin (OFLX)	0 (0.0)	0 (0.0)	62 (100.0)	≤12	13-15	≥16
Aminoglycoside	Gentamicin (CN)	3 (4.8)	21 (33.9)	38 (61.3)	≤12	13-14	≥15
	Kanamycin (K)	0 (0.0)	62 (100.0)	0 (0.0)	≤13	14-17	≥18
	Neomycin (N)	0 (0.0)	50 (80.6)	12 (19.4)	≤12	13-16	≥17
Tetracylines	Doxycycline (DO)	5 (8.1)	57 (91.9)	0 (0.0)	≤12	13-15	≥16
	Tetracycline (TET)	4 (6.5)	45 (72.6)	13 (21.0)	≤14	15-18	≥19
	Minocycline (MH)	0 (0.0)	58 (93.5)	4 (6.5)	≤14	15-18	≥19
Macrolides	Erythromycin (E)	0 (0.0)	0 (0.0)	62 (100.0)	≤13	14-22	≥23
	Medemycin (MAG)	0 (0.0)	29 (46.8)	33 (53.2)	≤13	14-17	≥18
Lincosamides	Clindamycin (DA)	5 (8.1)	10 (16.1)	47 (75.8)	≤14	15-20	≥21
Chloramphenicols	Chloramphenicol (C)	7 (11.3)	0 (0.0)	55 (88.7)	≤12	13-17	≥18
Sulfonamides	Chemitrim (SXT)	4 (6.5)	4 (6.5)	54 (87.1)	≤12	13-16	≥17
Glycopeptides	Vancomycin (VA)	37 (59.7)	25 (40.3)	0 (0.0)	≤9	10-11	≥12
Polymyxins	Polymyxin B (PB)	0 (0.0)	19 (30.6)	43 (69.4)	≤8	9-11	≥12
Nitrofurans	Furazolidone (FT)	0 (0.0)	28 (45.2)	34 (54.8)	≤14	15-16	≥17

^*^R, resistant; I, intermediate resistance; S, susceptibility.

**Table 4:** Multiple antimicrobial resistance (MAR) pattern of *V. parahaemolyticus*.

Resistance pattern	Isolate	Frequency of occurrence	Number of antibiotics	MAR index
AM, C	14	1	2	0.07
AMP, PRL	33	1	2	0.07
AMP, VA	23, 34	2	2	0.07
CXM, VA	1	1	2	0.07
OX, VA	41	1	2	0.07
AMP, CE, CL	87	1	3	0.1
AMP, CXM, VA	11	1	3	0.1
OX, AMP, PRL	22, 82, 91, 93, 94	5	3	0.1
AMP, C, PRL, VA	12	1	4	0.13
AMP, CE, OX, PRL	92	1	4	0.13
AMP, CXM, OX, PRL	29	1	4	0.13
AMP, CL, OX, PRL	62, 95	2	4	0.13
AMP, DO, OX, VA	5	1	4	0.13
AMP, OX, PRL, VA	16, 21, 26, 35, 54, 61, 85, 90	8	4	0.13
CE, CLVA, OX, PRL	83	1	4	0.13
C, OX, PRL, VA	13	1	4	0.13
AMP, CL, CXM, PRL, VA	2	1	5	0.17
AMP, CE, CL, OX, PRL	30, 81	2	5	0.17
AMP, CE, CXM, OX, PRL	89	1	5	0.17
AMP, CXM, DA, OX, PRL	76	1	5	0.17
AMP, CXM, OX, PRL, VA	15	1	5	0.17
AMP, OX, PRL, SXT, VA	36	1	5	0.17
CN, CXM, OX, PRL, VA	7	1	5	0.17
CXM, K, OX, PRL, VA	20	1	5	0.17
AMP, CE, CL, OX, PRL, VA	84	1	6	0.2
AMP, CXM, DO, OX, PRL, VA	28	1	6	0.2
AMP, KZ, OX, PRL, TE, VA	27	1	6	0.2
AMP, OX, PRL, SXT, TE, VA	25	1	6	0.2
CL, CN, CXM, OX, PRL, VA	9	1	6	0.2
AMP, C, CXM, DA, OX, PRL, VA	8	1	7	0.23

**Table 5:** Percentage of antimicrobial resistance genes for *V. parahaemolyticus* isolates (*n* = 62).

Antimicrobial class	Antimicrobial resistance genes	Percentage of *V. parahaemolyticus* (*n* = 62)
β-lactam	*bla*TEM	0
	*bla*OXA	0
	*bla*SHV	17.5
	*bla*CTX-M	42.9
Quinolone	*gyrA*	98.4
	*parC*	100
Aminoglycoside	*strA*	5.8
	*strB*	0
Tetracyline	*tetA*	41.3
	*tetB*	0
	*tetM*	11.1
Chloramphenicol	*catI*	33.3
	*catII*	0
	*catIII*	54
	*catIV*	11.1
Integrase	*intI1*	20.6
	*intI2*	0
	*intI3*	0
I integron variable region	BOX	9.5

**Table 6:** Plasmid pattern of *V. parahaemolyticus* from aquatic products.

Plasmid pattern	Isolates of *V. parahaemolyticus*
P1: 33 kb	1, 8, 20, 30, 36, 84, 89, 90, 91, 93, 94, 95
P2: 33 kb; 15 kb	14, 16, 21, 23, 34, 35, 41, 82, 83, 85, 87, 92
P3: 33 kb; 5 kb	33
P4: 33 kb; 18 kb; 15 kb	29, 54, 61, 62, 76
P5: 33 kb; 15 kb; 7 kb; 1.5 kb	11
P6: 33 kb; 18 kb; 3 kb; 1 kb	22
P7: 33 kb; 15 kb; 3 kb; 0.75 kb; 0.5 kb	81
None detected	5, 7, 8, 9, 12, 13, 20, 25, 27, 28, 36
